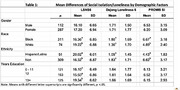# Social isolation and loneliness in diverse rural environments

**DOI:** 10.1002/alz70860_101249

**Published:** 2025-12-23

**Authors:** Juyoung Park, Christine L. Williams, Janet Holt, Lisa Ann Kirk Wiese

**Affiliations:** ^1^ University of Arizona College of Nursing, Tucson, AZ, USA; ^2^ Florida Atlantic University, C.E. Lynn College of Nursing, Boca Raton, FL, USA; ^3^ C.E. Lynn College of Nursing, Florida Atlantic University, Boca Raton, FL, USA

## Abstract

**Background:**

Social isolation (SI) is strongly associated with accelerated cognitive decline, yet comprehensive data are lacking among older adults in rural communities, particularly those who are racially/ethnically diverse and economically disadvantaged. The purpose of this study was to examine the impact of social isolation and loneliness on cognitive function in rural, community‐dwelling adults aged 45 and older.

**Method:**

Multiple validated measures were used to assess social isolation and loneliness, including the 4‐item NIH PROMIS Social Isolation Scale, the Lubben Social Network Scale‐6 (LSNS‐6), and the DeJong Loneliness Scale. These tools capture various dimensions of social connections, e.g., structural and functional aspects of social networks, perceived social support, and subjective feelings of loneliness. Tests of cognition, including the Montreal Cognitive Assessment (MoCA), were conducted to examine the relationship between these psychosocial factors and cognitive health.

**Results:**

Among 399 participants, MoCA and LSNS‐6 scores showed a low positive correlation (*r* = .24, *p* < .001), suggesting greater social engagement is associated with better cognitive function. The DeJong Loneliness Scale and PROMIS Social Isolation measures had significant low negative correlations with MoCA scores (*r* = ‐.13, *p* = .01 and *r* = ‐.17, *p* < .001, respectively), suggesting that higher social isolation and loneliness were associated with lower cognitive performance. No significant correlations were observed with self‐reported memory or mCAIDE score (ADRD risk). A low negative correlation was found between age and loneliness (*r* = ‐.10, *p* = .047), but no significant correlations emerged between social isolation measures and age or years residing in the study communities. Significant mean differences were observed between at least one social isolation measure and education level, race, and ethnicity, but not gender. Notably, ethnicity and race were significantly correlated with all three social isolation measures. Hispanic/Latino and White participants scored higher on social connectedness (LSNS‐6) and reported lower levels of loneliness and isolation.

**Conclusion:**

This study emphasizes the importance of examining relationships among race, ethnicity, and social engagement and impacts on brain health. Future research will explore potential mechanisms through which social isolation may mediate cognitive decline across racial and ethnic groups.